# Trimethylamine-N-oxide (TMAO) as Novel Potential Biomarker of Early Predictors of Metabolic Syndrome

**DOI:** 10.3390/nu10121971

**Published:** 2018-12-13

**Authors:** Luigi Barrea, Giuseppe Annunziata, Giovanna Muscogiuri, Carolina Di Somma, Daniela Laudisio, Maria Maisto, Giulia de Alteriis, Gian Carlo Tenore, Annamaria Colao, Silvia Savastano

**Affiliations:** 1 Dipartimento di Medicina Clinica e Chirurgia, Unit of Endocrinology, Federico II University Medical School of Naples, Via Sergio Pansini 5, 80131 Naples, Italy; giovanna.muscogiuri@gmail.com (G.M.); daniela.laudisio@libero.it (D.L.); dealteriisgiulia@gmail.com (G.d.A.); colao@unina.it (A.C.); sisavast@unina.it (S.S.); 2 Department of Pharmacy, University of Naples “Federico II”, Via Domenico Montesano 49, 80131 Naples, Italy; giuseppe.annunziata@unina.it (G.A.); maria.maisto@unina.it (M.M.); giancarlo.tenore@unina.it (G.C.T.); 3 IRCCS SDN, Napoli Via Gianturco 113, 80143 Naples, Italy; cdisomma@unina.it

**Keywords:** trimethylamine N-oxide (TMAO), obesity, visceral adiposity index (VAI), fatty liver index (FLI), metabolic syndrome (MetS)

## Abstract

There is a mechanistic link between the gut-derived metabolite trimethylamine-N-oxide (TMAO) and obesity-related diseases, suggesting that the TMAO pathway may also be linked to the pathogenesis of obesity. The Visceral Adiposity Index (VAI), a gender-specific indicator of adipose dysfunction, and the Fatty Liver Index (FLI), a predictor of non-alcoholic fatty liver disease (NAFLD), are early predictors of metabolic syndrome (MetS). In this cross-sectional observational study, we investigated TMAO levels in adults stratified according to Body Mass Index (BMI) and the association of TMAO with VAI and FLI. One hundred and thirty-seven adult subjects (59 males; 21–56 years) were enrolled. TMAO levels were detected using HPLC/MS analysis. Homeostatic Model Assessment of Insulin Resistance (HoMA-IR), VAI and FLI were included as cardio-metabolic indices. TMAO levels increased along with BMI and were positively associated with VAI and FLI, independently, on common potential covariates. The most sensitive and specific cut-offs for circulating levels of TMAO to predict the presence of NAFLD-FLI and MetS were ≥8.02 µM and ≥8.74 µM, respectively. These findings allow us to hypothesize a role of TMAO as an early biomarker of adipose dysfunction and NAFLD-FLI in all borderline conditions in which overt MetS is not present, and suggest that a specific cut-off of TMAO might help in identifying subjects at high risk of NAFLD.

## 1. Introduction

TMAO is a small, organic, gut microbiota-derived metabolite, which is emerging as a new potentially important cause of increased atherosclerosis and cardiovascular risk [1,2,3]. Circulating levels of TMAO increase after the gut microbial metabolism of dietary L-carnitine and phosphatidylcholine-rich foods, including red meat, eggs, dairy products, which are common nutrients of the Western diet. Very recently we reported a novel association between circulating levels of TMAO and the Mediterranean diet in healthy normal-weight adults, with a clear gender difference in this association [4]. The metabolic pathway of TMAO includes the digestion of these amines from gut microbiota with the production of trimethylamine (TMA), which is then converted to TMAO via flavin-monooxygenase-3 (FMO3) in the liver [5,6]. The interplay between dietary composition, gut microbiota and microbe-generated metabolites has been intensely investigated [7,8].

Several studies have shown a mechanistic link between TMAO, inflammatory pathways [9] atherosclerosis, type 2 diabetes mellitus (T2DM), and cardiovascular diseases (CVD) [1,2,3,6,10,11]. Namely, circulating levels of TMAO and its metabolites (choline and betaine) are associated with atherosclerosis risks in both humans and mice [1]. Among the pro-atherosclerotic mechanisms proposed for TMAO there are the inhibition of the reverse cholesterol transport, although this effect was reported in animal studies only [6], and the enhancement of human platelet hyperresponsiveness and thrombosis potential [10]. A systematic review and meta-analysis by Schiattarella et al. [2] demonstrated that in humans there is a positive dose-dependent association between circulating levels of TMAO and increased cardiovascular risk and mortality [2]. Nevertheless, in this metanalysis, the population samples were not divided according the BMI classes. More recently, Kanitsoraphan et al. [3] reported that in patients with T2DM higher circulating levels of TMAO were significantly associated with higher overall mortality by 2.07- to 2.7-fold, also after adjustment for BMI [3]. Recently, the strong association between gut microbiota and either obesity and obesity-related diseases in humans on the one hand, and the association between the TMAO pathway and cardio-metabolic diseases on the other, suggested that the TMAO pathway may be also mechanistically linked to the pathogenesis of obesity. Schugar et al. [11] reported that plasma TMAO levels were linked to with obesity traits in the different inbred strains of mice receiving a high-fat diet and suggested that the TMAO-generating pathway is linked to obesity and energy metabolism [11], although scientific evidence to support this association in humans has not yet been provided. Only Randrianarisoa et al. [12] reported that TMAO correlated positively with BMI, insulin resistance, visceral fat mass, and liver fat content [12]. In addition, Chen et al. [13] showed positive associations of the circulating TMAO levels and two of its nutrient precursors, choline, and betaine, with the presence and severity of NAFLD, the hepatic manifestation of the MetS [14], in a large sample of hospital- and community-based Chinese adults [13].

Nevertheless, controversy remains over the exact role of TMAO in the pathogenesis of MetS, a constellation of cardio-metabolic risk factors, including central obesity, hypertriglyceridemia, low high-density lipoprotein (HDL) cholesterol, hyperglycemia, and hypertension [15], which predispose T2DM and CVD [16], according to the definition of nascent MetS [17]. Very recently, a clinical study investigating several biogenic amines in urine, including TMAO in a sample of patients with MetS showed that these subjects presented higher levels of TMAO compared with their counterparts without MetS [18]. In addition, a further study investigating unselected white patients undergoing coronary angiography for the evaluation of suspected coronary artery disease showed a positive correlation between TMAO and MetS [19]. However, this association was lost after adjusting for impaired kidney function and poor metabolic control in this population sample.

On the other hand, this association was not found in a sample of patients with nascent MetS (without CVD or T2DM), but not with the commonly used surrogate index of insulin resistance, i.e., the HoMA-IR [9]. Again, in these recent studies, the population samples were not divided according to BMI classes.

The VAI, a gender-specific indicator of adipose distribution and dysfunction [20], and the FLI, an accurate predictor of the NAFLD [21], are two surrogate indices of cardio-metabolic risk and are linked to the inflammatory pathways [22,23]. Both indices are based on simple anthropometric and metabolic parameters, and are strictly correlated with MetS, representing early predictors of MetS [20,24]. VAI has been counted as an effective marker to assess insulin resistance and metabolic disturbances that contribute to CVD in primary-care non-obese subjects, with specific cut-off values depending on the population of interest [20]. Also, FLI has been proposed as a marker of insulin resistance [21] and recently the clinical utility of FLI as a predictor of incident T2DM has been reported [25]. As three of the variables making up VAI (waist circumference (WC), plasma triglycerides (TG), and HDL cholesterol) and FLI (BMI, WC, and TG) are used as continuous variables, and while they are dichotomically expressed in the criteria for MetS, VAI and FLI might represent useful indicators of early cardio-metabolic risk in all borderline conditions in which overt MetS is not present [22].

Considering the lack of evidence in humans of a progressive increase of TMAO levels across BMI classes and to gain further insight into the levels of TMAO in the setting of obesity, in this study we aimed to investigate the circulating levels of TMAO in a sample of the adult population stratified according to BMI. In addition, considering the still controversial role of TMAO in the pathogenesis of MetS and the predictive value of VAI and FLI as easy and early indicators of MetS, we investigated the association of circulating levels of TMAO with VAI and FLI and hypothesized that this association could serve as a biological marker of early metabolic derangement in subjects with overweight and obesity.

## 2. Materials and Methods

### 2.1. Design and Setting

This is a cross-sectional observational study carried out at the Department of Clinical Medicine and Surgery, Unit of Endocrinology, University Federico II, Naples (Italy), from January 2017 to August 2018. The work has been carried out in accordance with the Code of Ethics of the World Medical Association (Declaration of Helsinki) for experiments involving humans, and it has been approved by the Ethical Committee of the University of Naples “Federico II” Medical School (n. 173/16). The purpose of the protocol was explained to all the study participants, and written informed consent was obtained. This trial was registered at http://register.clinicaltrials.gov/. Unique identifier: NCT03060811.

### 2.2. Population Study

Recruitment strategies included a sample of 330 adult Caucasians subjects (20–63 years) of both genders consecutively enrolled among patients of our outpatient clinic, hospital volunteers, and employees from the same geographical area around Naples, Italy). All female subjects were non-pregnant and non-lactating. A full medical history, including drug use, was collected.

**Figure 1:**
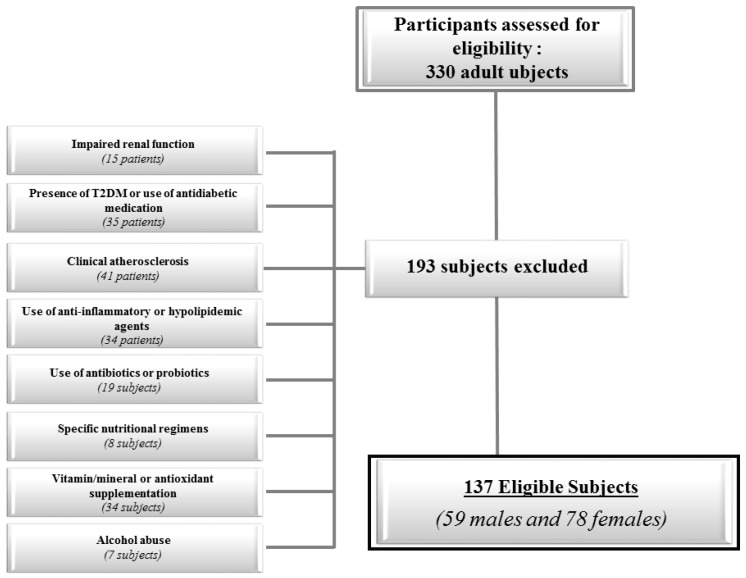
Flow chart of the studied subjects.

To increase the homogeneity of the subject sample, we included only adults of both genders with the following criteria of exclusion:

- Impaired renal function (normal values: estimated glomerular filtration rate ≥ 90 mL/min/1.73 m^2^ calculated by chronic kidney disease epidemiology collaboration equation; CKD EPI) (15 patients)
- Presence of T2DM (defined by criteria of the American Diabetes Association as follows: basal plasma glucose level ≥ 126 mg/dL on two occasions, or glycated hemoglobin (HbA1c) ≥ 6.5% (≥48 mmol/mol) on two occasions, or both at the same time (35 patients). Participants on antidiabetic medication were considered to have T2DM [26].
- Clinical atherosclerosis (coronary artery disease, peripheral vascular disease, CVD) (41 patients)
- Current therapy with anti-inflammatory drugs, statins and other hypolipidemic agents (34 patients);
- User of antibiotics or probiotics within 2 months of recruitment (19 subjects);
- Specific nutritional regimens, including vegan or vegetarian diets (eight subjects);
- Vitamin/mineral or antioxidant supplementation (34 subjects);
- Alcohol abuse according to the Diagnostic and Statistical Manual of Mental Disorders (DSM)-V diagnostic criteria (eleven subjects);

The flow chart of the study subjects is shown in Figure 1.

### 2.3. Lifestyle Habits

Lifestyle habits, including smoking and physical activity level, have been investigated as follows: subjects smoking at least one cigarette per day were considered current smokers, while former smokers were the subjects who stopped smoking at least one year before the interview. Remaining participants were defined as non-smokers. Physical activity levels were expressed according to whether the participant habitually engaged at least 30 min/day of aerobic exercise (YES/NO).

### 2.4. Anthropometric Measurements and Blood Pressure

Measurements were performed between 8 and 12 a.m. All subjects were measured after an overnight fast. The measurements were made in a standard way by the same operator (a nutritionist experienced in providing nutritional assessment and body composition). At the beginning of the study, all anthropometric measurements were taken with subjects wearing only light clothes and without shoes. In each subject, weight and height were measured to calculate the BMI [weight (kg) divided by height squared (m^2^), kg/m^2^]. Height was measured to the nearest 0.5 cm using a wall-mounted stadiometer (Seca 711; Seca, Hamburg, Germany). Body weight was determined to the nearest 0.1 kg using a calibrated balance beam scale (Seca 711; Seca, Hamburg, Germany). BMI was classified according to the World Health Organization (WHO)’s criteria with normal weight: 18.5–24.9 kg/m^2^; overweight, 25.0–29.9 kg/m^2^; grade I obesity, 30.0–34.9 kg/m^2^; grade II obesity, 35.0–39.9 kg/m^2^; grade III obesity ≥ 40.0 kg/m^2^ [27]. WC was measured to the closest 0.1 cm using a non-stretchable measuring tape at the natural indentation or at a midway level between the lower edge of the rib cage and the iliac crest if no natural indentation was visible, as per the National Center for Health Statistics (NCHS) [28]. In all subjects Systolic Blood Pressure (SBP) and Diastolic Blood Pressure (DBP) were measured three times, two min apart, with a random zero sphygmomanometer (Gelman Hawksley Ltd., Sussex, UK) after the subject had been sitting for at least 10 min. The average of the second and third reading was recorded.

### 2.5. Determination of Circulating Levels of TMAO

TMAO serum levels were measured in samples stored at −80  °C. A previous study indicated that, under these conditions, TMAO is stable for several years [29]. The quantification of circulating TMAO levels has been performed using the method described by Beale and Airs [30], and reported in our previous study [4], with slight modifications. Briefly, serum proteins were precipitated with methanol (serum:methanol, 1:2, *v*/*v*); samples were vortex-mixed for 2 min, centrifuged at 14,000 g for 10 min (4 °C) [31] and supernatants were collected and analyzed by High-Performance Liquid Chromatography-Mass Spectrometry (HPLC-MS) method. Both HPLC-MS conditions and method optimization were performed in accordance with Beale and Airs [30]. The HPLC system Jasco Extrema LC-4000 system (Jasco Inc., Easton, MD, USA) was coupled to a single quadrupole mass spectrometer (Advion ExpressIonL CMS, Advion Inc., Ithaca, NY, USA) equipped with an Electrospray ionization (ESI) source, operating in positive ion mode. The chromatographic separation was performed with a Luna HILIC column (150 × 3 mm, 5 µm particles) in combination with a guard column (HILIC), both supplied by Phenomenex (Torrance, CA, USA).

### 2.6. Assay Methods

Samples were collected in the morning between 8 and 10 a.m., after an overnight fast of at least 8 h and stored at −80 °C until being processed. All biochemical analyses including fasting plasma glucose, total cholesterol, fasting plasma TG, Alanine Transaminase (ALT), Aspartate Aminotransferase (AST), and γ-Glutamyltransferase (γGT) were performed with a Roche Modular Analytics System in the Central Biochemistry Laboratory of our Institution. Low-Density Lipoprotein (LDL) cholesterol and HDL cholesterol were determined by a direct method (homogeneous enzymatic assay for the direct quantitative determination of LDL and HDL cholesterol). Fasting insulin levels were measured by a solid-phase chemiluminescent enzyme immunoassay using commercially available kits (Immunolite Diagnostic Products Co., Los Angeles, CA, USA). The intra-assay coefficients of variations (CV) was <5.5%, as already widely reported in our previous studies [32,33,34,35,36].

### 2.7. Cardio-Metabolic Indices

HoMA-IR was calculated according to Matthews et al. [37]. A value of HoMA-IR >2.5 was used as cut-off of insulin resistance. VAI score has been calculated by the following sex-specific formula, with TG levels expressed in mmol/L and HDL levels expressed in mmol/L:

Males: VAI = [WC/39.68 + (1.88 × BMI)] × (TG/1.03) × (1.31/HDL),(1)

Females: VAI = [WC/36.58 + (1.89 × BMI)] × (TG/0.81) × (1.52/HDL),(2)

Age-specific VAI cut-off values were used according to Amato et al. [22,38]. In detail, cut-offs in subjects aged ≤30, 31–42, 43–52, and 53–66 years old were 2.52, 2.23, 1.92, 1.93, respectively [22,38].

FLI was calculated with the formula:

[FLI = eL/(1 + eL) × 100, L = 0.953 × loge triglycerides + 0.139 × BMI + 0.718 × logeγGT + 0.053 × WC−15.745].(3)

FLI of 30 was considered as the cut-off value based on Bedogni’s criterion [21].

### 2.8. Criteria to Define MetS

According to the National Cholesterol Education Program Adult Treatment Panel (NCEP ATP) III definition, MetS is present if three or more of the following five criteria are met: WC ≥ 102 cm (men) or 88 cm (women), blood pressure ≥ 130/85 mmHg, fasting TG level ≥ 150 mg/dL, fasting HDL cholesterol level ≤ 40 mg/dL (men) or ≤50 mg/dL (women), and fasting glucose ≥ 100 mg/dL [39].

### 2.9. Dietary Assessment

As has been already widely reported in the literature [34,35,36,40], data were obtained during a face-to-face interview between the patient and a qualified nutritionist. Specifically, the dietary interview allowed the quantification of foods and drinks by using a photographic food atlas (≈1000 photographs) of known portion sizes to ensure accurate completion of the records [41]. On day 1, the diary nutritionists were trained to standardized protocols and provided participants with instructions on how to complete the diary at the health check, and asked participants to recall the previous day’s intake. Participants prospectively completed the remaining 6 days and returned the records to the nutritionist [42]. Data were processed using a commercial software (Terapia Alimentare Dietosystem^®^ DS-Medica, http://www.dsmedica.info). Considering quantities of foods consumed, the software can calculate the total energy intake, expressed in kilocalories (kcal).

### 2.10. Statistical Analysis

The data distribution was evaluated by Kolmogorov-Smirnov test and the abnormal data were normalized by logarithm. Skewed variables were back-transformed for presentation in tables and figures. Results are expressed as mean ± standard deviation (SD). The chi square (χ^2^) test was used to determine the significance of differences in frequency distribution of smoking habit, physical activity, and presence/absence of MetS. Differences according to gender, lifestyle habits, cardio-metabolic indices, and MetS were analyzed by Student’s unpaired *t*-test, while the differences among the classes of BMI were analyzed by ANOVA followed by the Bonferroni post-hoc test. The correlations between study variables were performed using Pearson *r* correlation coefficients and were estimated after adjusting for gender, BMI, smoking, physical activity, and total energy intake. Proportional Odds Ratio (OR) models, 95% Interval Confidence (IC), and R^2^ were performed to assess the association between gender, lifestyle habits, classes of BMI, cardio-metabolic indices, and MetS. In addition, two multiple linear regression analysis models (stepwise method), expressed as R^2^, Beta (β), and *t*, with circulating levels of TMAO as dependent variables were used to estimate the predictive value of: (a) cardio-metabolic indices; and (b) FLI and MetS. Receiver operator characteristic (ROC) curve analysis was performed to determine sensitivity and specificity, area under the curve (AUC), and IC, as well as cut-off values of circulating levels of TMAO in detecting FLI and MetS. Test AUC for ROC analysis was also performed. We wanted to show that AUC being 0.943 for a particular test is significant from the null hypothesis value 0.5 (meaning no discriminating power), so we entered 0.943 for AUC ROC and 0.5 for null hypothesis values. For α level we selected 0.05 type I error and for β level we selected 0.20 type II error. In these analyses, we entered only those variables that had a *p*-value < 0.05 in the univariate analysis (partial correlation). To avoid multicollinearity, variables with a variance inflation factor (VIF) >10 were excluded. Values ≤0.05% were considered statistically significant. Data were stored and analyzed using the MedCalc^®^ package (Version 12.3.0 1993–2012 MedCalc Software bvba—MedCalc Software, Mariakerke, Belgium).

## 3. Results

The study population consisted of 137 participants, 59 males and 78 females, aged 21–56 years. Current smokers were 49.6% (68 subjects). A moderate-intensity aerobic activity at least 5 days per week was reported in 42.3% (58 subjects). BMIs ranged from 19.6 to 58.8 kg/m^2^. Median values of HoMA-IR, VAI and FLI were 1.95 (0.1–15.16), 1.89 (0.60–13.85) and 75.30 (3.40–100.0), respectively. In particular, 64 subjects (46.7%) had HoMA-IR values higher than the cut-off. VAI was higher than sex- and age-specific cut-offs in 43.8% (60 subjects) and 59.9% (82 subjects) presented FLI values above the cut-off. MetS was present in 53 participants (38.7%).

In Table 1 we report the lifestyle habits, anthropometric measurements, blood pressure, metabolic profile, and cardio-metabolic indices in the total population grouped based on BMI categories. As shown in Table 1, no differences were observed in age (*p* = 0.292), while subjects with overweight and obesity exhibited statistical differences in all parameters compared with normal-weight subjects (*p* < 0.001). In particular, circulating levels of TMAO increased with the BMI classes, with the highest TMAO values in the class III obesity.

Circulating levels of TMAO according to gender, lifestyle habits, and cut-off of the cardio-metabolic indices are reported in Table 2. As reported in Table 2, circulating levels of TMAO were significantly higher in males (*p* = 0.015), among current smokers or physically inactive individuals (*p* < 0.001), and in subjects with cardio-metabolic indices higher than cut-offs (*p* < 0.001). In addition, circulating levels of TMAO were significantly higher in presence of MetS (*p* < 0.001).

### Correlation Analysis

The correlations between circulating levels of TMAO, age, components of the MetS, metabolic profile, cardio-metabolic indices, are summarized in Table 3. Apart from the age, circulating levels of TMAO show significant correlations with all metabolic parameters. After adjusting for gender, BMI, smoking, physical activity, and total energy intake, correlations with almost all the components of MetS and cardio-metabolic indices were still evident, as shown in Table 3.

The results of bivariate proportional OR model performed to assess the association of circulating levels of TMAO with quantitative variables are reported in Table 4. The highest circulating levels of TMAO are significantly associated with the severity of obesity (OR 9.59; *p* < 0.001), and insulin resistance (OR 2.82; *p* < 0.001). Moreover, the highest levels of TMAO are associated with the highest levels of VAI (OR 1.58; *p* < 0.001) and FLI (OR 4.31; *p* < 0.001), presence of MetS (OR 2.36; *p* < 0.001) and of all components of the MetS.

To compare the relative predictive power of the cardio-metabolic indices associated with the circulating levels of TMAO, we performed a multiple linear regression analysis using a model that included as HoMA-IR, VAI, and FLI. Using this model, FLI entered at the first step (*p* < 0.001), while HoMA-IR, VAI were excluded. To compare the relative predictive power of FLI and number of components of MetS associated with the circulating levels of TMAO, we performed a second multiple linear regression analysis model. Using this second model, FLI entered at the first step (*p* < 0.001), while the number of components of MetS were excluded. Results were reported in Table 5.

**Figure 2:**
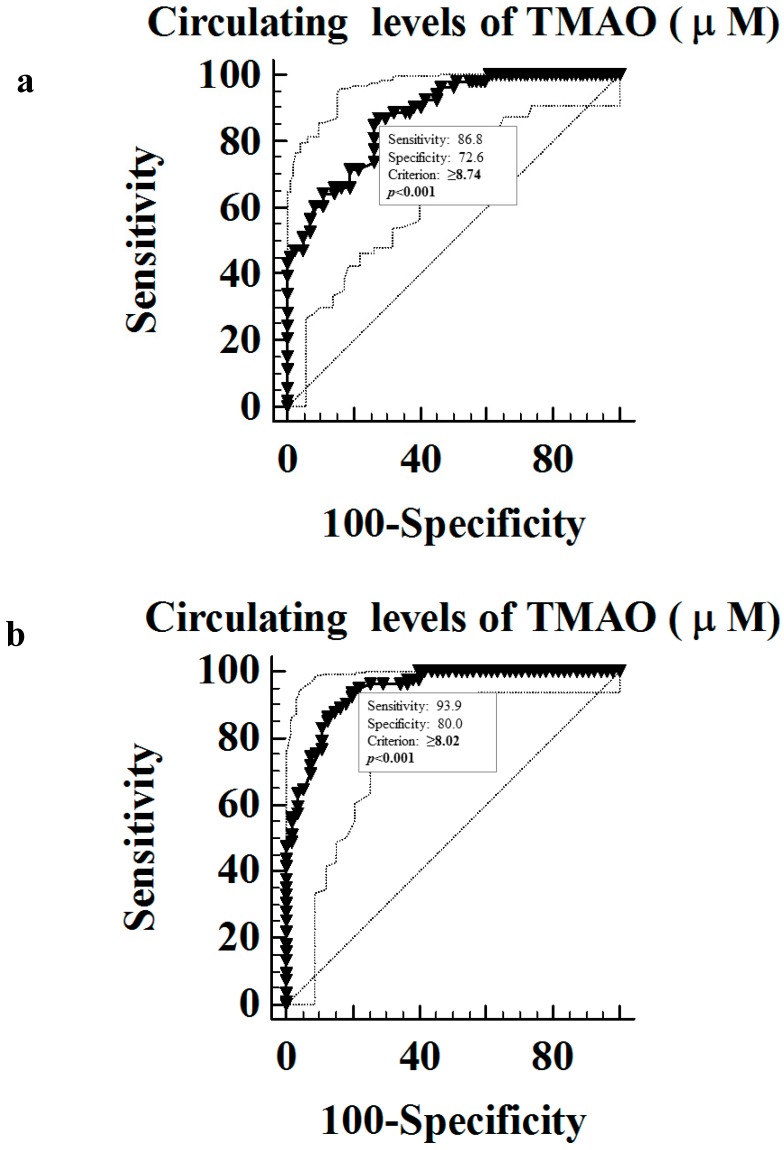
ROC for predictive values of circulating levels of TMAO in detecting FLI (**a**) and MetS (**b**). A *p*-value in bold type denotes a significant difference (*p* < 0.05).

A ROC analysis was then performed to determine the cut-off values of circulating levels of TMAO predictive of MetS and FLI. In particular, circulating levels of TMAO ≥8.74 µM (*p* < 0.001, AUC 0.876, standard error 0.029, 95% CI 0.808 to 0.926; Figure 2a), and circulating levels of TMAO ≥8.02 µM (*p* < 0.001, AUC 0.943, standard error 0.018, 95% CI 0.890 to 0.975; Figure 2b), could serve as thresholds for significantly increased risk of the presence of MetS and NAFLD, respectively.

## 4. Discussion

In this cross-sectional observational study, we evaluated the circulating levels of TMAO in a sample of adult population stratified according to categories of BMI. In addition, we investigated the association among circulating levels of TMAO and cardio-metabolic indices. The classification of the study population according to the BMI demonstrated that circulating levels of TMAO increased along with BMI. To the best of our knowledge, this is the first study that reported statistical differences in the circulating levels of TMAO across classes of BMI. Moreover, we confirmed the presence of a positive association of circulating levels of TMAO with MetS, the increasing number of its components, and HoMA-IR. Finally, a novel association was also reported among the circulating levels of TMAO and VAI and FLI, two surrogate indices of cardio-metabolic risk and early indicators of MetS, independently on common potential covariates.

There is a paucity of literature that has studied the relationship between TMAO and adiposity. A recent experimental study reported that both antisense oligonucleotide-mediated knockdown and genetic deletion of the TMAO-producing enzyme FMO3 protected mice against high-fat diet-induced obesity, thus highlighting a role of the gut microbe-driven TMA/FMO3/TMAO pathway in affecting specific transcriptional reprogramming in white adipocytes [11]. Of interest, in this study circulating levels of TMAO were positively associated with body weight, fat mass, mesenteric adiposity, and subcutaneous adiposity across the different mice-inbred strains; in addition, in cohorts of overweight or obese subjects with metabolic traits and different ethnicity the expression of FMO3 in liver biopsies was positively correlated with BMI and waist-to-hip ratio, and negatively correlated with the Matsuda Index, a measure of insulin sensitivity [11]. Consistent with these data, the findings of the present study show that there is a clear positive association of circulating TMAO levels and classes of BMI. Thus, besides its role as a risk factor for CVD and adverse event in risks subjects, emerging evidence suggests that gut microbiota-derived TMAO might represent per se a key environmental factor contributing to obesity and obesity-associated disorders.

VAI is a surrogate of adipocyte dysfunction independently correlated with insulin sensitivity, and cardio-metabolic risk in primary-care non-obese subjects [20]. In particular, VAI was proposed as a useful tool for the early detection of a condition of insulin resistance and cardio-metabolic risk before it develops into an overt MetS [38]. FLI is a surrogate marker of a fatty liver considered a screening tool to identify NAFLD, recognized as the liver manifestation of MetS, in subjects with insulin resistance and cardio-metabolic risk factors where ultrasound is unavailable [43]. In our population sample there was a positive association between circulating levels of TMAO and both indices. Of interest, the association of the circulating levels of TMAO with VAI and FLI was also shown independently on potential covariates, such as gender, BMI, smoking status, physical activity, and energy intake. In addition, among the cardio-metabolic indices included in this study, FLI, which incorporates BMI, WC, TG, and liver function, was a better predictor of TMAO variability than MetS per se. This finding was in line with the putative role of FLI as early predictor of MetS and likely reflects that the main site of expression of FMO3, the enzyme that metabolizes gut microbe-derived TMA to produce TMAO, is the liver. Based on ROC curve analysis, the most sensitive and specific cut-offs for circulating levels of TMAO to predict the presence of NAFLD-FLI and MetS were ≥8.02 µM and ≥8.74 µM, respectively. Experimental studies in mice fed with a high-fat diet showed that a high urinary excretion of TMAO was associated with insulin resistance and NAFLD in mice (129S6) prone to these diseases [44]. Miao et al. [45] found that liver insulin receptor knockout mice, characterized by a selective hepatic insulin resistance, have increased circulating TMAO levels associated with a strong up-regulation of the TMAO-producing enzyme FMO3 in the liver [45]. According to the proposed mechanism, TMAO may block the hepatic insulin signaling pathway, thereby exacerbating the impaired glucose tolerance, and promoting the development of fatty liver [46]. Turning to human studies, the above-mentioned study by Randrianarisoa et al. [12] reported a positive correlation between TMAO and liver fat content [12]. In addition, in a large sample of hospital and community-based Chinese adults, Chen et al. [13] showed positive associations of the circulating TMAO levels and two of its nutrient precursors, choline and betaine, with the presence and severity of NAFLD [13]. Choline can be oxidized to betaine, and betaine is generally regarded as safe for dietary ingestion due to its sparing effects on choline, a basic component for the synthesis of phosphatidylcholine, which in turn is necessary for promoting lipid exportation from the liver [47]. In this study, the authors suggested that TMAO may represent a possible risk factor for NAFLD due to its effect in altering the synthesis and transport of bile acids, with subsequent effects on lipid metabolism, intrahepatic triglycerides levels and glucose homeostasis [6]. More recently, Ntzouvani et al. [18] reported a strong positive association between liver function and a pattern of amino acids, which included TMAO, in a sample of Greek adult males with MetS [18].

The results of this study lend support to the evidence that circulating levels of TMAO were positively associated with body weight, thus concurring with an increased risk of developing MetS, through insulin resistance, adipocyte dysfunction, and fatty liver. In this context, it is tempting to hypothesize that circulating levels of TMAO could have a role of a biological marker of early metabolic derangement in subjects with overweight and obesity. Thus, it allows speculation on the possible beneficial and cost-effective effects to early address specific nutrition interventions aimed to reduce the excessive intake of TMAO precursors in subjects at risk of MetS and NAFLD.

Despite these very interesting results, the main limitation of this study is that the cross-sectional design does not allow identification of any causal association between the variables included. Thus, it is not possible to clearly determine the prognostic value of circulating levels of TMAO for predicting the early metabolic derangement in subjects with overweight and obesity. Second, although it is well known that the gut-derived origin of TMAO and dietary intake are important determinants of TMAO levels, we did not include in this study the gut microbiota and single-nutrient analysis. In particular, Kühn et al. [48] reported also that both dietary habits and the composition of the intestinal microbiota may be prone to changes, despite a certain stability of both factors during adulthood. On the other hand, Krüger et al. [49] reported that the large inter-individual variations TMAO levels have been mainly attributed to intestinal microbiota differences, while the influence of diet on fasting TMAO concentrations, albeit statistically significant, could be considered rather moderate. However, there is a large consensus that the study of gut microbiota is burdened by a high intra-individual variability that might hinder the interpretation of the results [48,50,51,52]. Third, we did not include the evaluation of markers of inflammation, such as C-reactive protein, or metabolic precursor of TMAO such as choline, betaine or carnitine, and liver expression of FMO3. However, several studies have shown that the associations between plasma TMAO and disease outcomes were independent of TMAO precursors [53,54,55]. In addition, the adverse effects of FMO3 on metabolic disease may be driven by factors other than TMAO, and the expression of the FMO3 or markers of inflammation encompassed the design of the present study. Fourth, we are aware that the liver biopsy is the gold-standard technique for identifying NAFLD. Liver biopsy is an invasive and costly procedure burdened with rare but potentially life-threatening complications. On the contrary, FLI has proved to represent an easy screening tool to identify NAFLD in patients with cardio-metabolic risk factors where ultrasound is unavailable [21,56,57], which is associated with reduced insulin sensitivity, and increased risk of T2DM, atherosclerosis, and cardiovascular disease [43]. Nevertheless, a major strength of this study is the good characterization of our sample population across BMI classes, with the exclusion of impaired renal function and T2DM known factors that can affect TMA metabolism, and likely with similar nutrient availability and food consumption pattern, as they were living in the same geographical area. Furthermore, we included of a variety of potential covariates, such as the total energy intake, to minimize the effect of confounding factors on the association of TMAO with adiposity. However, since complete understanding of TMAO biology is still lacking, the potential translation application of the results of this study to the clinical practice requires large-scale data investigating the beneficial and cost-effective effects of specific nutrition intervention aimed to avoid the excessive intake of TMAO precursors in subjects at risk of MetS and NAFLD. The main results of our study, compared to the results of the general literature, is reported in Table 6.

## 5. Conclusions

In conclusion, this study in a sample of adult subjects stratified according to their BMI: (i) reported a positive association between adiposity and circulating levels of TMAO; (ii) confirmed the positive association between circulating levels of TMAO and MetS; and (iii) further expanded the knowledge of the relationship of TMAO and MetS, as we reported novel associations between circulating levels of TMAO and two early indicators of MetS. In particular, in this study we demonstrated that FLI is more tightly associated with TMAO levels than the presence of MetS per se. These associations let us to hypothesize a role of TMAO as an early biomarker of NAFLD-FLI in all borderline conditions in which overt MetS is not present. Moreover, given the current performance of therapies for MetS, we suggest that a specific cut-off of TMAO might help in identifying subjects at high risk of NAFLD, who will require specific nutrition intervention strategies. Appropriate cross-validation studies in larger patient population samples are mandatory to validate the cut-off of TMAO for the identification of subjects at high risk of NAFLD-FLI.

## Figures and Tables

**Table 1:** Lifestyle habits, anthropometric characteristics, blood pressure, metabolic profile, cardio-metabolic indices, and total energy intake of participants grouped based on BMI categories.

Parameters	Normal Weight *n* = 34; 24.8%	Over Weight *n* = 29; 21.2%	Grade I Obesity *n* = 21; 15.3%	Grade II Obesity *n* = 15; 10.9%	Grade III Obesity *n* = 38; 27.7%	*p*-value
Lifestyle Habits						
Age (years)	35.71 ± 8.48	38.14 ± 7.58	38.24 ± 5.89	35.80 ± 8.20	35.00 ± 6.82	0.292
Smoking (yes)	16, 47.1%	19, 65.5%	4, 19.0%	2, 13.3%	22, 10.5%	*χ*^2^ = 19.21, ***p*** **<** **0.001**
Physical activity (yes)	22, 64.7%	11, 37.9%	3, 14.3%	5, 33.3%	4, 10.5%	*χ*^2^ = 27.85, ***p*** **<** **0.001**
Anthropometric measurement						
BMI (kg/m^2^)	23.01 ± 1.49	27.32 ± 1.43	32.41 ± 1.37	37.48 ± 1.56	46.99 ± 5.16	**<0.001**
WC (cm)	85.12 ± 10.13	94.30 ± 12.38	109.65 ± 8.14	118.81 ± 13.40	139.47 ± 15.15	**<0.001**
Blood pressure						
SBP (mmHg)	115.44 ± 8.01	121.21 ± 10.90	129.52 ± 10.83	131.00 ± 16.38	133.68 ± 11.79	**<0.001**
DBP (mmHg)	71.33 ± 6.07	75.68 ± 7.41	81.67 ± 6.77	86.33 ± 11.25	89.61 ± 9.25	**<0.001**
Metabolic profile						
Circulating levels of TMAO (µM)	3.62 ± 2.37	8.23 ± 0.67	9.03 ± 0.97	9.89 ± 0.85	11.53 ± 0.96	**<0.001**
Fasting Glucose (mg/dL)	83.65 ± 10.25	93.17 ± 13.10	96.47 ± 12.11	97.73 ± 11.00	121.87 ± 10.91	**<0.001**
Insulin (µU/mL)	2.66 ± 1.23	7.01 ± 5.35	10.69 ± 5.83	14.85 ± 9.65	31.29 ± 8.87	**<0.001**
Total cholesterol (mg/dL)	146.8 ± 20.28	176.69 ± 29.17	170.76 ± 20.85	206.87 ± 39.57	221.37 ± 33.58	**<0.001**
HDL cholesterol (mg/dL)	57.59 ± 7.53	50.21 ± 8.19	41.95 ± 13.28	39.60 ± 10.60	37.05 ± 9.42	**<0.001**
LDL cholesterol (mg/dL)	69.92 ± 23.15	101.43 ± 30.05	103.37 ± 16.67	134.49 ± 41.49	150.17 ± 38.54	**<0.001**
Triglycerides (mg/dL)	96.71 ± 26.96	125.24 ± 28.30	155.52 ± 65.23	163.87 ± 33.78	170.74 ± 70.88	**<0.001**
ALT (U/L)	23.26 ± 6.87	24.89 ± 9.06	38.14 ± 12.16	40.73 ± 17.87	41.39 ± 22.49	**<0.001**
AST (U/L)	20.44 ± 5.57	26.58 ± 6.67	36.83 ± 18.25	39.07 ± 14.10	41.00 ± 20.12	**<0.001**
γGT (U/L)	25.64 ± 6.62	26.52 ± 12.48	42.42 ± 19.71	44.47 ± 19.65	49.53 ± 27.20	**<0.001**
Cardio-metabolic indices						
HoMA-IR	0.55 ± 0.28	1.49 ± 0.96	2.51 ± 1.32	3.55 ± 2.31	9.52 ± 3.13	**<0.001**
VAI	1.28 ± 0.54	2.09 ± 1.27	3.42 ± 2.91	3.55 ± 1.97	3.77 ± 2.18	**<0.001**
FLI	19.89 ± 12.37	43.70 ± 21.36	79.39 ± 10.26	90.98 ± 6.97	98.36 ± 2.30	**<0.001**
Metabolic Syndrome						
MetS (number parameter)	0.18 ± 0.52	1.24 ± 1.02	2.33 ± 1.06	2.67 ± 1.40	3.68 ± 1.07	**<0.001**
MetS (presence)	0, 0	4, 13.8%	9, 42.9%	9, 60%	31, 81.6%	*χ*^2^ = 61.53, ***p*** **<** **0.001**
Nutritional parameters						
Total energy intake (kcal)	2084.79 ± 304.05	2249.14 ± 433.86	2423.33 ± 211.27	2658.67 ± 244.80	2966.45 ± 365.69	**<0.001**

A *p*-value in bold type denotes a significant difference (*p* < 0.05).

**Table 2:** Circulating levels of TMAO in the study population according to gender, lifestyle habits, cardio-metabolic indices, and MetS.

Parameters			Circulating Levels of TMAO (µM)	*p*-value
Gender		Males (n 59)	9.11 ± 3.09	**0.015**
		Females (n 78)	7.70 ± 3.28	
Smoking		Yes (n 68)	9.38 ± 2.63	**<0.001**
		No (n 69)	7.24 ± 3.49	
Physical activity		Yes (n 58)	6.41 ± 3.52	**<0.001**
		No (n 79)	9.69 ± 2.21	
HoMA-IR		> cut-off (n 64)	10.53 ± 1.62	**<0.001**
		< cut-off (n 73)	6.36 ± 3.01	
VAI		> cut-off (n 60)	10.08 ± 2.13	**<0.001**
		< cut-off (n 77)	6.92 ± 3.33	
FLI		> cut-off (n 82)	10.24 ± 1.56	**<0.001**
		< cut-off (n 55)	5.42 ± 3.00	
MetS (parameters)				
	WC	Yes (n 87)	9.88 ± 2.06	**<0.001**
		No (n 50)	5.56 ± 3.18	
	SBP/DBP	Yes (n 37)	10.49 ± 1.72	**<0.001**
		No (n 100)	7.50 ± 3.33	
	Fasting Glucose	Yes (n 43)	11.29 ± 1.22	**<0.001**
		No (n 94)	6.94 ± 2.98	
	HDL cholesterol	Yes (n 59)	10.13 ± 2.09	**<0.001**
		No (n 78)	6.93 ± 3.33	
	Triglycerides	Yes (n 45)	10.28 ± 2.20	**<0.001**
		No (n 92)	7.34 ± 3.27	
MetS (presence/absence)		Yes (n 53)	10.65 ± 1.62	**<0.001**
		No (n 84)	6.82 ± 3.17	

A *p*-value in bold type denotes a significant difference (*p* < 0.05).

**Table 3:** Correlations among circulating levels of TMAO with age, anthropometric characteristics, blood pressure, metabolic profile, cardio-metabolic indices, and nutritional parameter.

Parameters	Circulating Levels of TMAO (µM)		Circulating Levels of TMAO (µM)	
	Simple Correlation		After Adjusting	
	r	*p*-value	r	*p*-value
Age (years)	0.103	0.232	0.169	0.054
Anthropometric measurements				
BMI (kg/m^2^)	0.737	**<0.001**	-	-
WC (cm)	0.670	**<0.001**	−0.055	0.538
Blood pressure				
SBP (mmHg)	0.600	**<0.001**	0.273	**0.002**
DBP (mmHg)	0.532	**<0.001**	0.149	0.091
Metabolic profile				
Fasting Glucose (mg/dL)	0.656	**<0.001**	0.034	0.700
Insulin (µU/mL)	0.668	**<0.001**	0.202	**0.021**
Total cholesterol (mg/dL)	0.628	**<0.001**	0.236	**0.007**
HDL cholesterol (mg/dL)	−0.568	**<0.001**	−0.180	**0.041**
LDL cholesterol (mg/dL)	0.663	**<0.001**	0.356	**<0.001**
Triglycerides (mg/dL)	0.535	**<0.001**	0.224	**0.010**
ALT (U/L)	0.376	**0.001**	0.065	0.461
AST (U/L)	0.506	**<0.001**	0.176	**0.046**
γGT (U/L)	0.396	**0.001**	0.086	0.333
Cardio-metabolic indices				
HoMA-IR	0.699	**<0.001**	0.211	**0.016**
VAI	0.549	**<0.001**	0.255	**0.003**
FLI	0.820	**<0.001**	0.604	**<0.001**
Nutritional parameter				
Total energy intake (kcal)	0.592	**<0.001**	-	-

A *p*-value in bold type denotes a significant difference (*p* < 0.05).

**Table 4:** Bivariate proportional odds ratio model to assess the association between circulating levels of TMAO and gender, lifestyle habits, cardio-metabolic indices, and MetS.

Parameters		Circulating Levels of TMAO (µM)			
		OR	*p*-value	95% IC	R^2^
Gender		1.15	**0.015**	1.029–1.295	0.047
Smoking		1.26	**0.001**	1.110–1.423	0.108
Physical activity		0.67	**<0.001**	0.576–0.788	0.240
BMI categories					
	Normal weight	0.05	**0.001**	0.009–0.297	0.604
	Overweight	0.27	**<0.001**	0.011–1.121	0.209
	Grade I obesity	0.18	**<0.001**	0.010–0.099	0.237
	Grade II obesity	1.25	**<0.001**	0.995–1.565	0.033
	Grade III obesity	9.59	**<0.001**	3.946–23.344	0.561
HoMA-IR		2.82	**<0.001**	1.937–4.116	0.458
VAI		1.58	**<0.001**	1.308–1.912	0.248
FLI		4.31	**<0.001**	2.353–7.874	0.536
MetS (single parameters)					
	WC	1.88	**<0.001**	1.490–2.375	0.378
	SBP/DBP	1.64	**<0.001**	1.304–2.065	0.201
	Fasting Glucose	5.84	**<0.001**	3.161–10.804	0.538
	HDL cholesterol	1.61	**<0.001**	1.320–1.953	0.254
	Triglycerides	1.57	**0.001**	1.278–1.919	0.205
MetS (presence/absence)		2.36	**<0.001**	1.727–3.227	0.389

A *p*-value in bold type denotes a significant difference (*p* < 0.05).

**Table 5:** Multiple regression analysis models (stepwise method) with the circulating levels of TMAO as dependent variable to estimate the predictive value of: (a) cardio-metabolic indices; (b) FLI and MetS.

Parameters	Multiple Regression Analysis			
Model 1	R^2^	β	t	*p*-value
FLI	0.672	0.820	16.63	**<0.001**
Variables excluded: HoMA-IR and VAI				
Model 2				
FLI	0.469	0.685	9.2	**<0.001**
Variables excluded: MetS				

A *p*-value in bold type denotes a significant difference (*p* < 0.05).

**Table 6:** A summary table with the main results of our study compared to the results of the general literature.

Parameters	Methodology	Participants	Effects	Hypothesis	Studies	Concordance
**Nascent Metabolic Syndrome** **(MetS)**	Case-control clinical study	30 patients 20 controls	TMAO with a trend of positive correlation	TMAO levels rise only after MetS has advanced to the later stages including T2DM and/or CVD	[9]	Yes
						
**HoMA-IR**	Case-control clinical study	30 patients 20 controls	TMAO not significantly correlated	No major role for TMAO in glucose metabolism or insulin sensitivity	[9]	No
	Intervention Program	220 subjects	A negative correlation between circulating TMAO levels insulin sensitivity	In obese, hyperglycemic humans FMO3 expression and TMAO levels are increased in hepatic insulin resistance.	[12]	Yes
						
**NAFLD**	Experimental study	Mouse strain 129S6, documented for its susceptibility to IR or NAFLD	Mice 129S6 fed with a high-fat diet showed a high urinary excretion of TMAO associated with insulin resistance and NAFLD	A high-fat diet reduces the conversion and the bioavailability of choline by microbiota, causing NAFLD	[44]	Yes
	Experimental study	Male ob/ob mice and their lean, wild-type C57BL/6J controls	Liver insulin receptor knockout mice with selective hepatic insulin resistance have increased circulating TMAO levels associated with a strong up-regulation of the TMAO-producing enzyme FMO3 in the liver	TMAO may block the hepatic insulin signaling pathway promoting the development of fatty liver	[11]	Yes
						
	Case-control study (CCS) and cross-sectional study (CSS)	60 adult patients and 35 controls for CCS 1.628participants for CSS	TMAO is an independent risk marker for NAFLD in humans. in both the CCS and CSS studies	TMAO decreases the total bile acid pool size and influences the hepatic triglycerides levels, as a potential risk factor for fatty liver disease	[13]	Yes
	Intervention Program	220 subjects	A positive correlation between circulating TMAO levels and liver fat content	Fasting levels of TMAO are regulated by hepatic FMO3	[12]	Yes
	Cross-sectional study	One hundred middle-aged men	A strong positive association between liver function and a pattern of amino acids, which included TMAO	A pattern of amino acids, included TMAO, are regulated by liver enzymes	[18]	Yes
						

Abbreviations: MetS, Metabolic Syndrome; TMAO, Trimethylamine N-oxide; T2DM, Type 2 Diabetes Mellitus; CVD, Cardiovascular Diseases; HoMA-IR, Homeostatic Model Assessment Insulin Resistance; FMO3, Flavin-containing Monooxygenases; NAFLD, non-alcoholic fatty liver disease; IR, insulin Resistance; CCS, Case-control Study; CSS, Cross-sectional Study.

## Abbreviations

TMAO	Trimethylamine-N-oxide
TMA	Trimethylamine
FMO3	Flavin-monooxygenase-3
T2DM	Type 2 Diabetes Mellitus
CVD	Cardiovascular diseases
MetS	Metabolic Syndrome
HDL	High-density Lipoprotein
HoMA-IR	Homeostatic Model Assessment of Insulin Resistance
BMI	body mass index
VAI	Visceral Adiposity Index
FLI	Fatty Liver Index
NAFLD	Non-alcoholic Fatty Liver Disease
WC	Waist Circumference
TG	Triglycerides
SBP	Systolic Blood Pressure
DBP	Diastolic Blood Pressure
ALT	Alanine Transaminase
AST	Aspartate Aminotransferase
γGT	γ-Glutamyltransferase
LDL	Low-Density Lipoprotein
SD	Standard Deviation
OR	Odds Ratio
IC	Interval Confidence
ROC	Receiver Operator Characteristic
AUC	Area Under Curve.
